# Predictive factors of early recurrence after R0 resection of hilar cholangiocarcinoma: A single institution experience in China

**DOI:** 10.1002/cam4.2052

**Published:** 2019-03-13

**Authors:** Hai‐Jie Hu, Yan‐Wen Jin, Anuj Shrestha, Wen‐Jie Ma, Jun‐Ke Wang, Fei Liu, Ya‐Yun Zhu, Rong‐Xing Zhou, Parbatraj Regmi, Nan‐Sheng Cheng, Fu‐Yu Li

**Affiliations:** ^1^ Department of Biliary Surgery West China Hospital of Sichuan University Chengdu Sichuan Province China; ^2^ Department of General Surgery Gandaki Medical College Pokhara Nepal; ^3^ Department of Liver Surgery Zhongshan Hospital Fudan University Shanghai China

**Keywords:** early recurrence, hilar cholangiocrcinoma, prognosis, survival

## Abstract

Prediction of early postoperative recurrence is of great significance for follow‐up treatment. However, there are few studies available that focus on high‐risk factors of early postoperative recurrence or even the definition the exact time of early recurrence for hilar cholangiocarcinoma. Thus, we aimed to examine the optimal cut‐off value for defining the early in patients with R0 resection of hilar cholangiocarcinoma and to investigate prognostic factors associated with early recurrence. Two hundred and fifty‐eight patients with R0 resection of hilar cholangiocarcinoma between 2000 and 2015 were included. The minimum *P* value approach was used to define the optimal cut‐off of early recurrence. The prognostic factors associated with early recurrence were investigated. The optimal cut‐off value for dividing patients into early and non‐early recurrence groups after R0 resection of hilar cholangiocarcinoma was 12 months. Sixty‐two patients were recorded as early recurrence, and the remaining 196 patients were labeled as non‐early recurrence. Multivariate logistic regression analysis indicated lymph node metastasis (OR = 2.756, 95% CI 1.409‐5.393; *P* = 0.003), poor differentiation (OR = 1.653; 95% CI 1.040‐2.632; *P* = 0.034), increased postoperative CA 19‐9 levels (OR = 1.965, 95% CI 1.282‐3.013; *P* = 0.002), neutrophil‐to‐lymphocyte ratio > 3.41 (OR = 5.125, 95% CI 2.419‐10.857; *P* < 0.001) and age > 60 years (OR = 2.018, 95% CI 1.032‐3.947; *P* = 0.040) were independent determinants of early and non‐early recurrence. Poor differentiation (HR = 2.609, 95% CI 1.600‐4.252; *P* < 0.001), Bismuth classification type III/IV (HR = 2.510, 95% CI 1.298‐4.852; *P* = 0.006) and perineural invasion (HR=2.380, 95% CI 1.271‐4.457; *P* = 0.007) were independent factors of overall survival in the subgroup of patients who developed early recurrence. The optimal cut‐off value for dividing early recurrence after R0 resection of hilar cholangiocarcinoma was 12 months. Tumor differentiation, Bismuth classification, and perineural invasion were independent factors of overall survival in the subgroup of patients with early recurrence. Patients with risk factors should be monitored closely after curative surgery.

## 1 INTRODUCTION

Hilar cholangiocrcinoma (HCCA), accounting for approximately 40%‐60% of all bile duct cancers, is a devastating tumor arising from the conjunctive region of right and left hepatic bile ducts.^1^ Given the locally advanced nature of this disease, the capability of achieving R0 resection margin is often restricted to limited series. The curative resectability rate was reported as low as 18%‐42% and the majority of patients are only eligible for palliative therapy.^2^, ^3^, ^4^ Witzigmann et al^5^ reported an R0 resection rate of 23% in 184 HCCA patients, while Hu et al^6^ reported an R0 resection rate of 37.0% in 814 HCCA patients. Curative management including bile duct resection, major hepatic resection, caudate lobectomy, lymph node dissection, proper hepato‐enteric anastomosis and vascular resection and reconstruction, remains the cornerstone of treatment option for HCCA.^7^, ^8^ HCCA, however, is pathologically characterized by the advanced biological behavior of early neural infringement, vascular invasion, and early lymph node and caudate lobe metastasis, resulting in high postoperative recurrence rate and poor survival outcome even after curative resection.^3^, ^9^ The 5‐year disease‐free survival (DFS) and overall survival (OS) still remain very dismal, which is still presenting as a challenge for hepatobiliary surgeons.^1^

High rate of recurrence tends to be associated with poor survival outcome. Recently, the term “early recurrence” has been broadly applied in various tumors,^10^, ^11^, ^12^, ^13^ which, however, is merely used in HCCA and has not been clearly defined. Neutrophil‐to‐lymphocyte ratio (NLR) and platelet‐lymphocyte ratio (PLR) were currently available independent prognostic factors with predictive value of recurrence for several tumors.^1^, ^14^, ^15^, ^16^, ^17^, ^18^ Furthermore, the relationship of preoperative and clinicopathologic factors with early recurrence has also not been discussed in HCCA.

Thus, we aimed to identify the best cut‐off point between the early and non‐early recurrence in patients receiving R0 resection of HCCA in our high‐volume center employing the “minimum‐value” method. In addition, we investigated preoperative and clinicopathologic factors associated with early and non‐early recurrence, and then we further examined the prognostic factors for overall survival in the subgroup patients with early recurrence.

## 2 MATERIALS AND METHODS

### 2.1 Patient selection

A summary of 258 patients who underwent R0 resection for HCCA between 2000 and 2015 at the West China Hospital of Sichuan University were included. Patients without R0 surgery or those with intrahepatic cholangiocarcinoma involving the hepatic portal, distal cholangiocarcinoma, ampullary carcinomas, and gallbladder cancer were definitely excluded. The histologically diagnosed adenocarcinoma was identified in all selected patients.

### 2.2 Preoperative management and surgical procedures

All patients were evaluated with systematic inspection and elaborative imaging examination prior to surgery. Preoperative biliary drainage (PBD) was performed in patients with obstructive jaundice or cholangitis (n = 143), and portal vein embolization (PVE) was performed in patients with suspected remnant liver volume less than 30% (n = 38), approximately 4‐6 weeks ahead of the scheduled operation date. Surgical procedures were finally determined and conducted according to preoperative multidisciplinary team (MDT) discussion and intraoperative exploration. Intraoperative ultrasound and intraoperative frozen‐section histology examination were used to guide resection.

### 2.3 Follow‐up

All patients underwent closely surveillance at outpatient department with the regular (approximately 1‐2 months) test of liver function, tumor markers, ultrasonography for recurrence. Computed tomography (CT) or magnetic resonance imaging (MRI) was performed for those with highly suspicious tumor recurrence or those who were at the scheduled time of 6 months after surgery.

### 2.4 Defining recurrence and early recurrence

Recurrence was confirmed by any new lesions detected by CT or MRI; the subsequent follow‐up was closely monitored to detect disease progression. Patients were classified as local (including liver resection margin and liver hilum) and distant (including para‐aortic lymph nodes, peritoneum, intrahepatic, extra‐abdominal) recurrence based on the initial recurrence pattern. Patients were divided into early and non‐early recurrence groups; the minimum *P* value method (using the log‐rank test for the OS) was employed to determine the best dividing point of early and non‐early recurrence based on the OS.^10^, ^19^

### 2.5 Statistical analysis

Analysis of continuous variables was conducted using the Student's test or the Mann‐Whitney test while categorical variables were analyzed using Chi‐square or Fisher's exact test. The receiver operating characteristic (ROC) curve analysis was performed to identify the best cut‐off point of NLR, PLR, and tumor markers with tumor recurrence. Univariate and multivariate logistic regression models were used to check the potential preoperative and clinicopathologic factors associated with early and non‐early recurrence. Kaplan–Meier approach was used for survival evaluation; differences were compared between groups using the log‐rank test. A *P* value < 0.05 was considered statistically significant. All data were presented using the SPSS version 16.0 (SPSS Inc. Chicago, IL, USA).

## 3 RESULTS

### 3.1 Patients’ characteristics

A total of 258 patients with R0 resection of HCCA were included with a median age of 60 years (range: 26‐82), of which 153 patients were male and the remaining 105 patients were female. The specific surgical procedures were as follows: hilar bile duct resection (32 cases), left hemihepatectomy (113 cases), right hemihepatectomy (72 cases), left trisegmentectomy (23 cases), right trisegmentectomy (10 cases), and mesohepatectomy (eight cases). Caudate lobe was conventionally removed (221 cases), except for some earlier cases of type I papillary carcinoma. Combined vascular resection was conducted on 67 patients.

**Figure 1:**
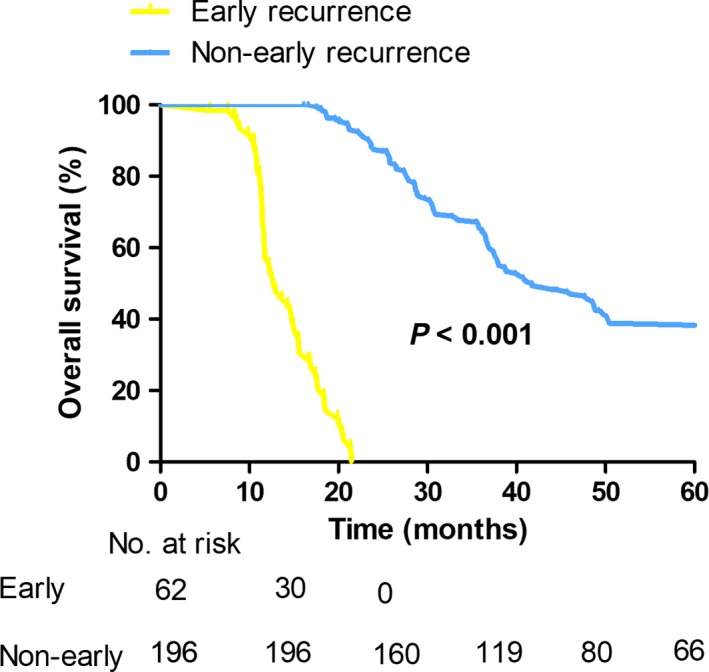
Kaplan‐Meier curves comparing survival status based on early and non‐early recurrence in patients with R0 resection of hilar cholangiocarcinoma (*P* < 0.001)

In the 258 patients, the median follow‐up time was 42.4 months. Total R0 resection rate in our center during the analyzed period of time was 34.5% (including those advanced disease without surgery). R0 resection provided the best OS with a median survival time of 36.7 months, and the 1‐, 3‐, and 5‐year survival rates of 91%, 51%, and 30%, respectively. The median DFS was 22.8 months, with the 1‐, 3‐, and 5‐year DFS rates of 78%, 29%, and 14%, respectively. The optimal cut‐off point for distinguishing patients with early recurrence from those with non‐early recurrence and thus divide patients into two sets based on the maximum difference in OS after initial recurrence was 12 months (*X* ^2^ = 353.15; *P* < 0.001; Figure 1) by taking the minimum *P* value method; 62 patients were subsequently recorded as early recurrence, and the remaining 196 patients were labeled as non‐early recurrence. The initial recurrence patterns for patients with early recurrence included local (n = 27) and distant recurrence (n = 35); which included liver resection margin (n = 8), liver hilum (n = 19) for local recurrence and Para‐aortic lymph nodes (n = 10), peritoneum (n = 5), intrahepatic (n = 12), and extra‐abdominal (n = 8) for distant recurrence.

### 3.2 Factors associated with early recurrence

Then, we identified the preoperative factors associated with early and non‐early recurrence (Table 1), we found that patients with early recurrence tended to have larger median age compared with those with non‐early recurrence (63 years vs 60 years; *P* = 0.025); patients over 60 were also more likely to have early tumor recurrence than those under sixty (58.1% vs 45.4%), although statistical did not reached (*P* = 0.082). More importantly, higher ratio of neutrophil‐to‐lymphocyte (4.40 vs 3.10; *P* < 0.001) and platelet‐to‐lymphocyte (250.20 vs 187.32; *P* < 0.001) were also manifested in patients with early recurrence compared with those non‐early recurrence patients. When examined as categorical variables, patients with NLR > 3.41 were more likely to have early recurrence compared with patients who had NLR ≤ 3.41 (40.0% vs 9.0%; *P* < 0.001). Of the 62 patients who had early recurrence, 38 patients (61.3%) were labeled as PLR > 238.8, while in the remaining 196 patients with non‐early recurrence, only 53 (27%) of them were classified as PLR > 238.8 (*P* < 0.001). In fact, the ROC analysis also identified that NLR > 3.41 can predict early recurrence with a sensitivity of 80.6% and specificity of 61.7%; PLR > 238.8 can also predict early recurrence with a sensitivity and specificity of 61.3% and 73.0%, respectively. In addition, a likelihood of lower preoperative albumin and lymphocytes levels and higher neutrophils levels were also witnessed in patients with early recurrence, although the *P* values did not reach statistical difference in comparison with non‐early recurrence patients, further statistical difference was also not obtained when examined as categorical variables (*P* > 0.05).

**Table 1:** Analysis of preoperative factors with early and non‐early recurrence

**Variables**	Early recurrence (n = 62)	Non‐early recurrence (n = 196)	*P* value
Age^a^	63.00 (56.00‐68.00)	60.00 (50.00‐67.00)	0.025
Gender/Male (%)	42 (67.7)	111 (56.6)	0.121
Preoperative CA 19‐9 level, U/mL^a^	288.55 (109.25‐527.08)	251.50 (67.44‐458.30)	0.094
Preoperative CA 125 level, U/mL^a^	20.61 (13.03‐25.50)	17.69 (12.33‐25.64)	0.542
Preoperative CEA level, ng/mL^a^	3.70 (2.58‐5.88)	3.16 (2.20‐4.84)	0.101
Preoperative TB level, umol/L^a^	225.05 (141.88‐278.33)	180.50 (102.28‐302.70)	0.102
Preoperative ALT level, U/L^a^	96.50 (52.75‐158.50)	95.00 (50.00‐169.50)	0.984
Preoperative AST level, U/L^a^	85.00 (58.75‐130.25)	87.00 (57.25‐140.25)	0.834
Preoperative Albumin level, g/L^a^	35.60 (31.75‐39.00)	36.90 (33.90‐40.28)	0.076
NLR^a^	4.40 (3.52‐5.34)	3.10 (2.75‐4.13)	<0.001
PLR^a^	250.20 (1.99‐2.88)	187.32 (146.55‐242.67)	<0.001
Preoperative tumor size, cm^a^	2.55 (2.00‐3.00)	2.50 (2.00‐3.00)	0.866
Preoperative Biliary drainage (%)	38 (61.3)	105 (53.6)	0.287
Bismuth‐Corlette classification (%)			
Type I and Type II	26 (41.9)	94 (48.0)	0.407
Type III and Type IV	36 (58.1)	102 (52.0)	

ALT, alanine aminotransferase; AST, aspartate transaminase; CA‐19‐9, carbohydrate antigenic determinant 19‐9; CA125, carbohydrate antigen 125; CEA, carcino embryonie antigen; NLR, neutrophil‐to‐lymphocyte ratio; PLR, platelet‐lymphocyte ratio; TB, total bilirubin.

a Parameters are presented as median (Interquartile range).

Further analysis of the clinicopathologic and postoperative factors with tumor recurrence showed that patients with lymph node metastasis (*P* < 0.001), vascular invasion (*P* = 0.009), perineural invasion (*P* = 0.003), poor differentiation (*P* = 0.001), and increased postoperative CA 19‐9 levels (*P* = 0.002) had higher rates of early recurrence (Table 2).

**Table 2:** Univariate analysis of clinicopathologic and postoperative factors with early and non‐early recurrence

Variables	Early recurrence (n = 62	Non‐early recurrence (n = 196)	*P* value
Lymph node metastasis			
No	24 (38.7)	137 (69.9)	<0.001
Yes	38 (61.3)	59 (30.1)	
Vascular invasion			
No	38 (61.3)	153 (78.1)	0.009
Yes	24 (38.7)	43 (21.9)	
Tumor size			
≤3 cm	42 (67.7)	152 (77.6)	0.119
>3 cm	20 (32.3)	44 (22.4)	
Perineural invasion			
No	28 (45.2)	130 (66.3)	0.003
Yes	34 (54.8)	66 (33.7)	
Tumor differentiation			
Poor	27 (43.5)	44 (22.4)	0.001
Moderate	26 (41.6)	87 (44.4)	
Well	9 (14.5)	65 (33.2)	
Caudate lobe resection			
No	12 (19.4)	25 (12.8)	0.196
Yes	50 (80.6)	171 (87.2)	
Postoperative complications			
No	46 (74.2)	134 (64.8)	0.170
Yes	16 (25.8)	71 (35.2)	
Postoperative CA 19‐9 levels			
Increased	24 (38.7)	39 (19.9)	0.002
Decreased ≤ 50%	22 (35.5)	64 (32.7)	
Decreased > 50%	16 (25.8)	93 (47.4)	

CA‐19‐9, carbohydrate antigenic determinant 19‐9. Other factors included postoperative CEA and CA 125 levels.

To estimate the independent contributing determinants to the early and non‐early recurrence, a multivariate logistic regression model was carried out (Table 3). It indicated that lymph node metastasis (OR = 2.756, 95% CI 1.409‐5.393; *P* = 0.003), poor differentiation (OR = 1.653; 95% CI 1.040‐2.632; *P* = 0.034), increased postoperative CA 19‐9 levels (OR = 1.965, 95% CI 1.282‐3.013; *P *= 0.002), NLR > 3.41 (OR = 5.125, 95% CI 2.419‐10.857; *P* < 0.001) and age > 60 years (OR = 2.018, 95% CI 1.032‐3.947; *P* = 0.040) were independent determinants of early recurrence in patients with R0 resection of HCCA.

**Table 3:** Variables associated with early and non‐early recurrence in multivariate logistic analysis

Variables	Odds ratio	95% CI	*P* value
Lymph node metastasis	2.756	1.409‐5.393	0.003
Tumor differentiation	1.653	1.040‐2.632	0.034
Increased postoperative CA 19‐9 levels	1.965	1.282‐3.013	0.002
NLR > 3.41	5.125	2.419‐10.857	<0.001
Age > 60 years	2.018	1.032‐3.947	0.040

CA‐19‐9, carbohydrate antigenic determinant 19‐9; NLR, neutrophil‐to‐lymphocyte ratio.

### 3.3 Predictors of OS in the patients with early recurrence

**Figure 2:**
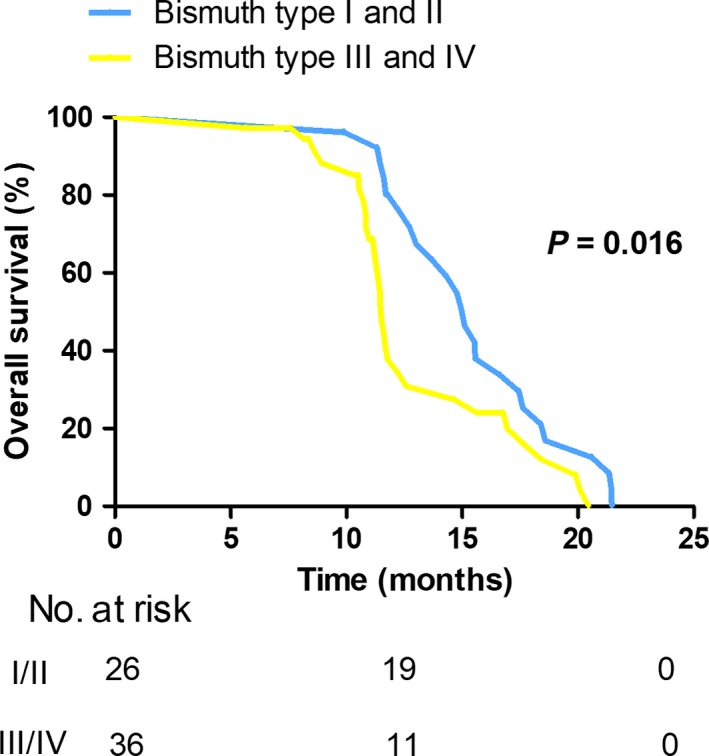
Kaplan‐Meier curves comparing survival status based on Bismuth classification in selected patients with early recurrence (*P* = 0.016)

**Figure 3:**
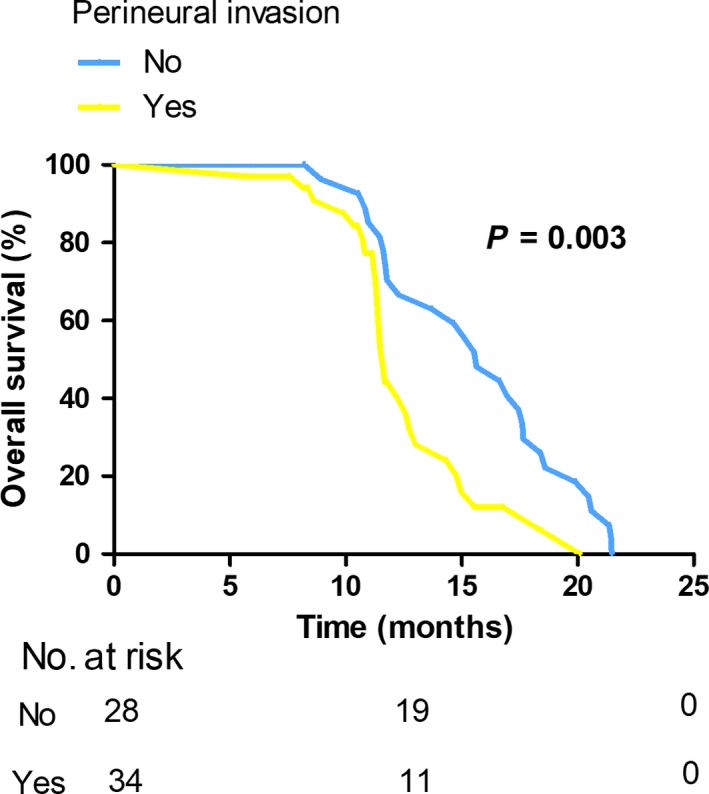
Kaplan‐Meier curves comparing survival status based on the presence of perineural invasion in selected patients with early recurrence (*P* = 0.003)

**Figure 4:**
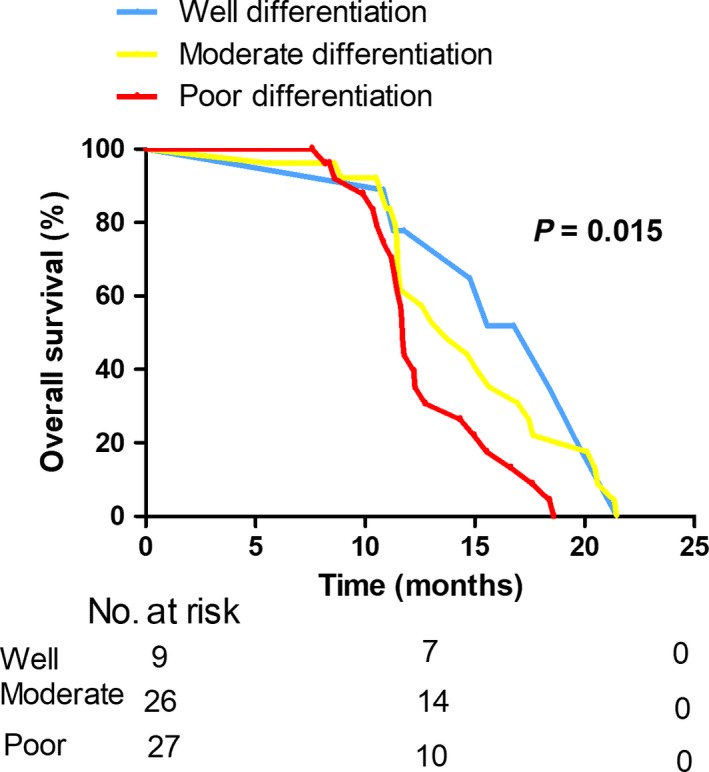
Kaplan‐Meier curves comparing survival status based on tumor differentiation in selected patients with early recurrence (*P* = 0.015)

Additionally, we examined potential predictive factors of OS in patients with early recurrence (Table 4). The univariate analysis demonstrated patients with tumor size > 3 cm (*P* = 0.018), Bismuth classification type III and IV (*P* = 0.018; Figure 2), perineural invasion (*P* = 0.003; Figure 3), poor differentiation (*P* = 0.006; Figure 4), CA 125 > 35 U/mL (*P* = 0.034) had poorer overall survival outcomes. In the multivariate analysis, poor differentiation (HR = 2.609, 95% CI 1.600‐4.252; *P* < 0.001), Bismuth classification type III and IV (HR = 2.510, 95% CI 1.298‐4.852; *P* = 0.006) and perineural invasion (HR = 2.380, 95% CI 1.271‐4.457; *P* = 0.007) were independent factors of OS in patients with early recurrence.

**Table 4:** Univariate and multivariate analysis of tumor factors associated with overall survival in the subgroup of patients with early recurrence

Tumor factors	Univariate			Multivariate		
	Hazard Ratio	95% CI	*P* value	Hazard Ratio	95% CI	*P* value
Age > 60 years	1.079	0.612‐1.903	0.793			
Male gender	0.722	0.396‐1.313	0.285			
Tumor >3 cm	2.202	1.146‐4.230	0.018			
Bismuth classification type III and IV	2.002	1.126‐3.561	0.018	2.510	1.298‐4.852	0.006
CA 19‐9 > 200 U/mL	0.717	0.332‐1.547	0.396			
ALT > 50 U/L	0.732	0.388‐1.379	0.334			
AST > 40 U/L	1.103	0.468‐2.602	0.823			
CA 125 > 35 U/mL	2.465	1.069‐5.683	0.034			
CEA > 3.4 ng/mL	1.224	0.692‐2.165	0.487			
Preoperative biliary drainage	1.373	0.777‐2.428	0.275			
Caudate lobe resection	1.531	0.734‐3.197	0.256			
Major hepatectomy	1.357	0.728‐2.527	0.336			
AJCC T stage	0.857	0.487‐1.506	0.591			
Positive nodal status	0.886	0.506‐1.550	0.672			
Poor differentiation	1.846	1.196‐2.849	0.006	2.609	1.600‐4.252	<0.001
Vascular invasion	1.235	0.697‐2.188	0.470			
Perineural invasion	2.435	1.344‐4.413	0.003	2.380	1.271‐4.457	0.007

CA‐19‐9, carbohydrate antigenic determinant 19‐9; CA125, carbohydrate antigen 125; CEA, carcino embryonie antigen; ALT, alanine aminotransferase. AST, aspartate transaminase; AJCC, American Joint Committee on Cancer.

## 4 DISCUSSION

Early recurrence has been previously reported as an adverse factor for poor survival outcome in various cancers, including the pancreatic carcinoma, the hepatocellular carcinoma and the gastric cancer.^10^, ^12^ HCCA has the characteristics of early postoperative recurrence and poor survival outcome. Despite the fact that some current studies have explored the recurrence patterns of HCCA patients after R0 resection, little has been known about the predictive factors of early recurrence. In the current study, the best cut‐off point for differentiating early recurrence from non‐early recurrence by means of the minimum *P* value method, our result found the best cut‐off point in differentiating early and non‐early recurrence, was 12 months, based on which, patients were divided into two groups: early (n = 62) and non‐early recurrence (n = 196). The initial recurrence patterns of the 62 patients with early recurrence showed a majority of patients (54.6%) had distant recurrence, of which intrahepatic metastasis was the most common type (n = 12); and for patients with local recurrence, liver hilum relapse showed the largest percentage with 19 patients detected.

Currently, there are still debates about the impact of the systemic inflammation factors on postoperative survival outcome, some have reported that systemic inflammation factors like NLR and PLR are associated with poor survival outcome and tumor recurrence.^14^, ^20^, ^21^ Dumitrascu et al reported that patients with the NLR level larger than median (median value: 3.3) had worse disease‐free survival.^22^ While Okuno et al identified a NLR cut‐off point of 3 and found NLR was not correlated with survival outcomes. Thus, studies with larger sample size are required. In the current study, ROC analysis was employed to identify the optimal (with the largest Youden index) predictive point of NLR and PLR for predicting early and non‐early recurrence. We demonstrated the ideal NLR cut‐off value was 3.41, with a sensitivity of 80.6%, and a specificity of 61.7%; the optimal PLR cut‐off value of 238.8 was also calculated with a sensitivity of 61.3% and a specificity of 73.0%, respectively. Furthermore, the subsequent univariate and multivariate models verified the predictive value of PLR and NLR for predicting tumor recurrence. Our results showed that patients with PLR > 238.8 or patients with NLR > 3.41 obviously had higher incidence of early recurrence, although PLR > 238.8 failed to maintain statistical difference as an independent factor of early recurrence in multivariate analysis. Meanwhile, patients with early recurrence also had larger median value of NLR level (4.40 vs 3.10; *P* < 0.001). Taken together, preoperative NLR level can serve as a predictive indicator of tumor recurrence; patients with higher level of preoperative NLR tend to have early tumor recurrence even after R0 resection.

Tumor differentiation and lymph node status are important prognostic factors for patients with HCCA, poor tumor differentiation and lymph node metastasis are disease progress biomarkers and adverse biologic factors for poor survival outcome.^6^, ^23^, ^24^, ^25^ In the present study, compared with patients with poor tumor differentiation, patients with well tumor differentiation had lower rate of early recurrence (38.0% vs 12.2%; *P* = 0.001). Our data also evidenced the theory that lymph node metastasis was correlated with an increased likelihood of developing early tumor recurrence (35.1% vs 11.8%; *P* < 0.001). We also noted that tumor differentiation and lymph node metastasis remained to be predictive factors by multivariate analysis after controlling for other competing factors. Importantly, poor differentiation was also an independent factor for predicting the OS in the selected patients of early recurrence. Thus, given the notion that tumor differentiation and lymph node status were predictors of early recurrence, it was not surprisingly that patients with poor tumor differentiation and lymph node metastasis would also have poorer survival outcome.

CA 19‐9 level is an important biomarker for HCCA. Patients with higher level of preoperative CA 19‐9 levels are more likely to have a poorer survival outcome and lower resectability rate.^4^, ^26^ In our current study, preoperative CA 19‐9 level failed to predict early recurrence whereas postoperative CA 19‐9 level was identified as a prognostic factor for early recurrence. Patients were enrolled into three groups based on the preoperative CA 19‐9 levels and postoperative CA 19‐9 levels within the first three months after surgery; then, patients were labeled as increased (n = 63), decreased ≤ 50% (n = 86) and decreased > 50% (n = 109). We noted that patients with increased postoperative CA 19‐9 levels conferred to a higher probability of early recurrence. Therefore, in viewing of the fact that elevated postoperative CA 19‐9 level was associated with early recurrence, postoperative CA19‐9 levels should be monitored closely so as to help detect early tumor relapse at some level.

Interestingly, our current study also noted that age > 60 years could also have the predictive value for selecting patients with early recurrence. A report conducted by Anderson et al^27^ indicated that a majority of patients diagnosed with cholangiocarcinoma tended to be elderly. There are also some studies concerning the prognostic effect of age on survival outcomes, with aging populations having impaired survivals.^28^, ^29^ We noted that patients with age > 60 years had an early recurrence rate of 28.8%, higher than 19.5% of those with age ≤ 60 years. Given the notion that age > 60 years remained as a detrimental factor for predicting early recurrence, patient age should be taken into consideration before surgery.

Then, further focus was concentrated on factors contributing to OS in patients with early recurrence. Our data suggested that Bismuth type III and IV, tumor differentiation and perineural invasion were independent predictors of OS in this section. Perineural invasion was observed in 28%‐100% of the patients resected for HCCA.^8^ Some previous studies also reported perineural invasion was associated with poor survival outcome.^30^, ^31^, ^32^, ^33^ In the current study, perineural invasion was noted to have an adverse effect on postoperative survival in the selected cases of early recurrence. Compared with non‐perineural invasion (15.63 months), perineural invasion contributed to a poor median OS of 11.60 months (*P* = 0.003). In aggregate, these data suggest perineural invasion has the ability to affect OS in patients with early recurrence. The prognostic value of Bismuth classification on survival is controversial.^23^ In the present study, our results revealed a significant survival difference between type III/IV and I/II Bismuth classification in patients with tumor recurrence within the first year; a less advanced Bismuth classification conferred to a relatively well median OS of 15.10 months while type III/IV carcinoma resulted in an inferior median OS of 11.50 months (*P* = 0.016). Thus, we believe advanced Bismuth classification may lead to poor survival outcome in patients with early recurrence.

To the best of our knowledge, this was the first time to analyze early recurrence and the factors associated with it in patients with R0 resection of HCCA. Our current study did have some limitations based on the retrospective nature and the variety of optimal cut‐off points of early and non‐early recurrence in different centers. Further studies are needed so as to better predict the capacity of the early and non‐early recurrence for HCCA.

In conclusion, the optimal cut‐off value for dividing early recurrence after R0 resection of HCCA was 12 months. Lymph node metastasis, poor differentiation, increased postoperative CA 19‐9 levels, NLR > 3.41 and age > 60 years were independent determinants of early and non‐early recurrence in patients with R0 resection of HCCA. Bismuth classification type III and IV, poor differentiation and perineural invasion were independent factors of OS in patients with early recurrence. Patients with risk factors should be monitored closely even after curative surgery.

## CONFLICT OF INTEREST

We declare that we have no conflict of interest.
